# Preparative Scale Resolution of Enantiomers Enables Accelerated Drug Discovery and Development

**DOI:** 10.3390/molecules22010158

**Published:** 2017-01-18

**Authors:** Hanna Leek, Shalini Andersson

**Affiliations:** 1 Respiratory, Inflammation and Autoimmunity, Innovative Medicines and Early Development Biotech Unit, AstraZeneca, Pepparedsleden 1, 431 83 Molndal, Sweden; hanna.leek@astrazeneca.com; 2 Cardiovascular and Metabolic Diseases, Innovative Medicines and Early Development Biotech Unit, AstraZeneca, Pepparedsleden 1, 431 83 Molndal, Sweden

**Keywords:** chiral resolution, chiral stationary phases, preparative chromatography, supercritical fluidchromatography, racemization, active pharmaceutical ingredient, drug discovery, drug development

## Abstract

The provision of pure enantiomers is of increasing importance not only for the pharmaceutical industry but also for agro-chemistry and biotechnology. In drug discovery and development, the enantiomers of a chiral drug depict unique chemical and pharmacological behaviors in a chiral environment, such as the human body, in which the stereochemistry of the chiral drugs determines their pharmacokinetic, pharmacodynamic and toxicological properties. We present a number of challenging case studies of up-to-kilogram separations of racemic or enriched isomer mixtures using preparative liquid chromatography and super critical fluid chromatography to generate individual enantiomers that have enabled the development of new candidate drugs within AstraZeneca. The combination of chromatography and racemization as well as strategies on when to apply preparative chiral chromatography of enantiomers in a multi-step synthesis of a drug compound can further facilitate accelerated drug discovery and the early clinical evaluation of the drug candidates.

## 1. Introduction

Stereochemistry plays a major role in drug discovery and the molecular mechanisms by which a chiral biological macromolecule selectively interacts with a drug compound, which may be an agonist, activator, antagonist, substrate, or inhibitor of the key function of the target in question, is of importance to understand [1]. Also, many of the processes involved in drug disposition, i.e., absorption, distribution, metabolism and excretion, involve a direct interaction with chiral biological macromolecules, e.g., transporters and enzymes, and following administration of a racemate the individual enantiomers may exhibit different properties that need to be taken into account when developing a novel drug [2]. Progress in chemical technologies associated with the synthesis, analysis and preparative-scale resolution of the enantiomers has greatly advanced drug discovery and development, and as a result, the potential significance of the differential pharmacodynamics, pharmacokinetic and toxicological properties of the individual enantiomers present in a racemate are appreciated and easier to evaluate [3].

At the discovery stage of drug development, when a large number of molecules with different compound properties are required in small quantities for initial testing, and where most compounds are only made once, asymmetric synthesis is not time- or cost-efficient. Further, both enantiomers are needed for biological testing, which would require two synthetic routes to be developed. Resolution of a racemate or enriched isomeric mixture has the advantage of providing both enantiomers and can be achieved using several methods including crystallization, enzymatic resolution, indirect chromatographic resolution and direct chromatographic resolution [4]. Development of a crystallization method or an enzymatic resolution can often be a time-inefficient approach for isolating small quantities of enantiomers. However, these methodologies in combination with chromatography and racemization of the undesired isomer deliver great efficiencies in the resolution of larger amounts of enantiomers [5,6].

The use of chromatographic resolution using chiral stationary phases (CSPs) has thus become the most time- and cost-effective approach for enantiomer resolution at the discovery stage in the pharmaceutical industry [7,8,9]. The development of CSPs goes back several decades and has been recently reviewed by Shen and Okamoto [10]. During the last decades, a large number of novel CSPs were developed and demonstrated enantioselectivity for a huge variety of compounds; however, only a limited number of these have been made commercially available. The availability of larger amounts of the required CSP is critical in that it allows a useful separation of isomers identified at an analytical scale on a specific CSP to be scaled up and thus enable preparative-scale separations of isomers in a limited time frame. Hence, the most commonly used CSPs in AstraZeneca laboratories which are commercially available at a kilogram scale are based on polymers such as polysaccharides [11] and cross-linked tartardiamides [12]. Other useful CSPs are the Pirkle-type Whelk-O1 phase [13] and the cinchona alkaloid–based weak anionic exchangers [14]. For the gram- to kilogram-scale amount of enantiomers required at the later stages of drug discovery and toxicological studies, chromatographic resolution via HPLC has been utilized for the past 25 years [15,16,17]. However, during the last decade the use of super critical fluid chromatography (SFC) has gained greater utility as a fast, cost-effective approach for the resolution of enantiomers [18,19]. In SFC the bulk of the solvent in the mobile phase is highly pressurized carbon dioxide (CO_2_). The low viscosity and high diffusivity of the SFC mobile phase allows higher flow rates relative to HPLC, resulting in shorter run times and increased efficiencies [20,21]. A major advantage of preparative SFC vs. preparative HPLC is the reduced solvent consumption and higher product concentrations post-chromatography, decreasing the time and energy expense for solvent removal and enantiomer isolation [22]. Also, the health and safety issues are highly improved by replacing solvents such as hexane or heptane used in normal-phase LC (NPLC) with the much less toxic CO_2_ [23]. We present case studies wherein the different technologies mentioned above for obtaining up-to-kilogram amounts of pure enantiomers have been utilized either as stand-alone technologies or in combination to deliver highly efficient processes that enable rapid access to high-purity material for biological testing.

## 2. Results

### 2.1. Identifying the Optimal Method for Preparative-Scale Chiral Separation

As the compound requirements for initial discovery testing are relatively small (<50 mg) and the number of different molecules large, the approach for chiral analytical method development must be both time- and compound-efficient [24,25,26]. The initial CSP and mobile phase evaluation is not designed to provide complete resolution, but to quickly differentiate between stationary phases and chromatographic conditions that afford separation and those that do not. In addition, the evaluation conditions need to encompass a range of compounds with varying physicochemical properties. SFC is the primary technique used in AstraZeneca’s laboratories and HPLC is only applied when SFC is unsuccessful. A focused SFC screen, containing four CSPs (Chiralpak AD, Chiralcel OJ, Whelk-O1 and Lux Cellulose 4) and two mobile phases (ethanol and 2-propanol), quickly and efficiently enables a resolution of ~80% of the screening compounds based on our internal database encompassing 2000 novel chiral compounds collected since 2013. The remaining 20% of the compounds are subject to further evaluation using the full battery of 13 stationary phases. For larger scale (multiple g–kg), an exhaustive evaluation is always undertaken to ensure that optimal conditions are used for the preparative separation with respect to solvent consumption, chromatographic throughput and timely delivery. In addition, the compound solubility in the mobile phase must be considered as this has a strong impact on the efficiency of the separation process. Further, the stability of the compound in the injection solvent and in the mobile phase is important to determine in order to minimize chemical degradation and/or racemization of both the racemate as well as the isolated enantiomers.

### 2.2. Case Study I: Application of SFC or NPLC in Enantiomeric Separations and Impact of Compound Solubility

For an oncology project with the aim to treat acute myeloid leukemia, the pure enantiomer of the optimized active pharmaceutical ingredient (API) at a 100 g scale was required in order to carry out the toxicology studies. In general, the aim is to reduce the consumption of expensive reagents and to avoid wasting valuable material and thus it is often favorable to perform the chiral separation as early as possible in the synthetic route, especially as the savings can be quite substantial at the kilogram scale. Here, this was done after the first four synthesis steps of a nine-step reaction to reach the final product, requiring the separation of 900 g of the racemic intermediate compound **A**. The compound is a weak base and consists of two atropisomers that arise from hindered rotation around a single bond.

**Figure 1:**
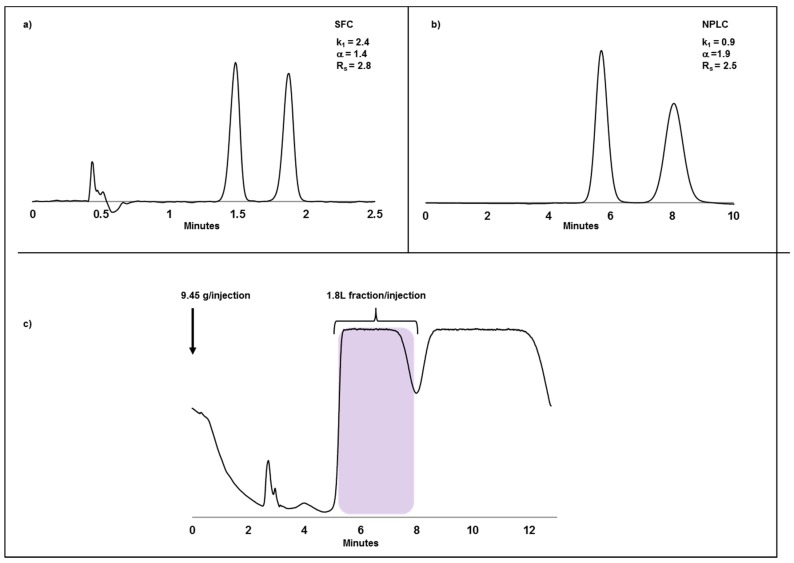
Chiral separation of a basic compound **A**. (**a**) Analytical SFC chromatogram using a Chiralpak IA 150 mm × 4.6 mm, 3 µm column. Mobile phase was 15% methanol/dichloromethane/diethyl amine 50/50/0.5 (*v*/*v*/*v* %) in CO_2_ at 40 °C, 120 bar and a flow rate of 4 mL/min; (**b**) Analytical NPLC chromatogram using a Chiralpak IA 250 mm × 4.6 mm, 20 µm column. Mobile phase was heptane/ethyl acetate/diethyl amine 50/50/0.5 (*v*/*v*/*v* %) at a flow rate of 1 mL/min; (**c**) Preparative chromatogram on Chiralpak IA 250 mm × 110 mm, 20 µm. 9.45 g (270 mg/mL dichloromethane) was injected every 13 min using heptane/ethyl acetate/diethyl amine 50/50/0.5 (*v*/*v*/*v* %) at a flow rate of 600 mL/min. The blue area corresponds to the product fraction, 1.8 L/injection each cycle.

One concern that had to be considered during method development was the limited solubility of compound **A**, especially in alcohols. Thus, alternative solvents that would enhance solubility in both HPLC and SFC eluents such as dichloromethane and ethyl acetate [27,28], only applicable on chemically immobilized chiral stationary phases, were explored [29,30]. Utilization of chlorinated solvent is always seen as a last resort as it has been linked to cancer in mammalians [31]. Further optimization showed useful analytical-scale separation in the SFC mode on the immobilized CSP Chiralpak IA which is based on amylose tris (3,5-dimethylphenylcarbamate) as a chiral selector immobilized onto silica (Figure 1a). Unfortunately, loadability studies showed distorted peaks even though dichloromethane was used as a solubility enhancer in the mobile phase. Furthermore, loadability was too low and only 150 mg could be injected in each cycle on a 3 cm column. In addition, an increased back pressure was observed over time, typically associated with precipitation on the column inlet frit [32]. Without sufficient solubility of the compound in the supercritical mobile phase, the separation was deemed to be less efficient to deliver for the drug project’s needs. Thus, a technology shift to NPLC was decided and due to the observed solubility issues in the SFC mode, only aprotic solvents were evaluated in the screening for optimized mobile phase conditions. The final method was identified to be Chiralpak IA using a heptane/ethyl acetate 60/40 mixture (Figure 1b). For large-scale separation, an 11 cm column was used at a flow rate of 600 mL/min with 9.45 g injected each cycle giving a fraction volume of around 1.8 L (Figure 1c). To maximize the amount of racemate processed per time unit, the stacked injection technique was used, i.e., the next injection was carried out before total elution from the on-going cycle. Even though the chromatography could not be run overnight due to large solvent consumption and limited mobile phase container volumes (200 L), the material was processed and delivered in time with an enantiomeric excess of 98.2% and a yield of 98%. The throughput was however low, reaching only 0.9 kg racemate/kg CSP/day and consuming 0.83 m^3^ of solvent mixture per kg of racemate chromatographed.

### 2.3. Case Study II: A Highly Efficient Chiral Separation of Kilogram Amounts of a Racemic Drug Compound on Chiralpak IC

In the development of a first-line anti-inflammatory therapy for asthma patients, the synthesis of the racemic API, compound **B**, an organic carboxylic acid, was outsourced and the first large batch (kg amount) required in the project was for early-stage toxicology testing. During the discovery phase, the chiral resolution was carried out early in the synthetic route which consisted of seven steps. However, it was shown that this compound was quite easily racemized during the subsequent synthesis steps and thus chiral chromatography would need to be re-applied again in a later synthesis step, leading to additional loss of precious material as well as time. To remove this risk, the synthetic route was changed and chiral chromatography was now performed for the API instead, with a subsequent crystallization step to obtain the desired polymorph of the (+)-enantiomer as a final step. Due to capacity issues at the contract research organization (CRO), the chiral chromatography and crystallization steps were carried out in-house at AstraZeneca.

**Figure 2:**
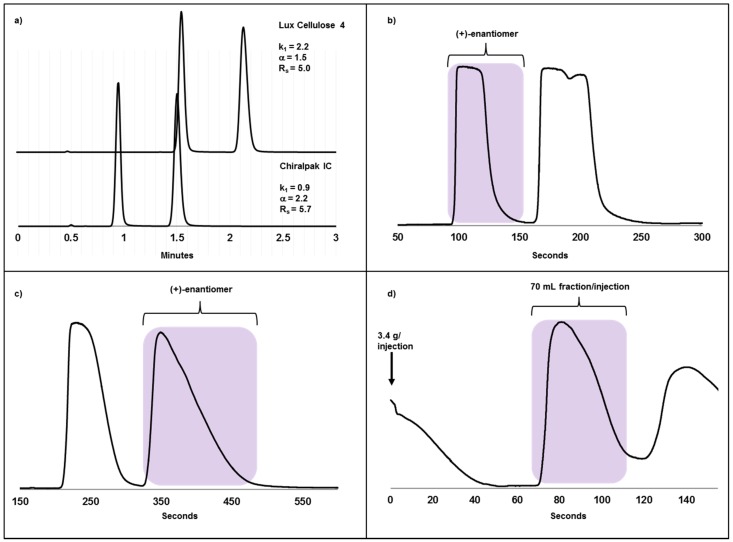
Chiral separation of compound **B**, a racemic carboxylic acid. (**a**) Analytical SFC chromatograms using 150 mm × 4.6 mm, 3 µm columns. Mobile phases were 20% methanol/formic acid 100/0.5 (*v*/*v* %) on Lux Cellulose 4 and 30% ethanol/formic acid 100/0.5 (*v*/*v* %) in CO_2_ at 40 °C, 120 bar and a flow rate of 4 mL/min; (**b**) Loadability study on Chiralpak IC. Racemate (600 mg, 200 mg/mL ethanol) was injected on a 250 mm × 30 mm, 5 µm column using 20% ethanol/formic acid 100/0.5 (*v*/*v* %) in CO_2_ at 40 °C, 120 bar and a flow rate of 150 g/min. The blue area corresponds to the product fraction, on this column the first eluting enantiomer; (**c**) Loadability study on Lux Cellulose 4. Racemate (600 mg, 200 mg/mL ethanol) was injected on a 250 mm × 30 mm, 5 µm column using 15% methanol/formic acid 100/0.5 (*v*/*v* %) in CO_2_ at 40 °C, 120 bar and a flow rate of 150 g/min. The blue area corresponds to the product fraction, on this column the second eluting enantiomer; (**d**) Preparative chromatogram on Chiralpak IC 250 mm × 50 mm, 5 µm. 3.4 g (200 mg/mL ethanol) was injected every 155 s using 20% ethanol/formic acid 100/0.5 (*v*/*v* %) in CO_2_ at 40 °C, 120 bar and a flow rate of 450 g/min. The blue area corresponds to the product fraction, 70 mL/injection each injection.

Prior to receiving the 2.5 kg batch from the CRO, compound **B** was evaluated on 13 different CSPs. Two of the stationary phases were identified as candidates for further optimization and loadability studies, namely Chiralpak IC and Lux Cellulose 4 which are both based on a cellulose derivate containing the tris (3,5-dichloro methyl phenyl carbamate) and the tris (4-chloro-3-methylphenylcarbamate) moieties, respectively. Chiralpak IC gave a higher α-value (2.2) and lower retention times than the Lux Cellulose CSP (α = 1.5) using the SFC mode and optimized mobile phase conditions, (Figure 2a). An additional advantage of choosing Chiralpak IC for the large-scale separation, apart from the shorter cycle times was that the desired enantiomer eluted first leading to smaller fraction volumes and thus higher productivity (Figure 2b,c).

A 250 mm × 50 mm column, using 20% ethanol in CO_2_ at 120 bar and 40 °C with a flow rate of 450 g/min, was used for the large-scale batch. As a result, 3.4 g racemate was resolved in 155 s, leading to a product fraction of less than 70 mL (Figure 2d), with about a 10-times higher product concentration compared with the previously described case I. To maintain a high level of safety for the operators, the entire chromatographic system including the evaporator was connected with stainless steel tubing and no handling of the compound was done after the racemate had been dissolved and placed in the feed tank. Reproducible and stable chromatography together with a low solvent consumption enabled a continuous operation and the enantiomers were resolved, evaporated and delivered within two days for toxicological evaluation. Since the product fraction was continuously evaporated, dry product was acquired shortly after the chromatographic step was finished. A total of 1.1 kg of the API was obtained in an enantiomeric excess of 99.9% and a yield of 96% for this step. This example, with a chromatographic throughput of 5.7 kg racemate/kg CSP/day, demonstrates many of the advantages of applying SFC for the separation of enantiomers. A low solvent consumption of 0.07 m^3^/kg racemate and an efficient evaporation process, as well as a contained sample handling, enabled this campaign to be delivered in a safe manner in a research laboratory.

### 2.4. Case Study III: A Combination of Chiral Chromatography and Racemization of the Undesired Isomer Gives a Highly Efficient Process for Obtaining a Single Enantiomer

**Figure 3:**
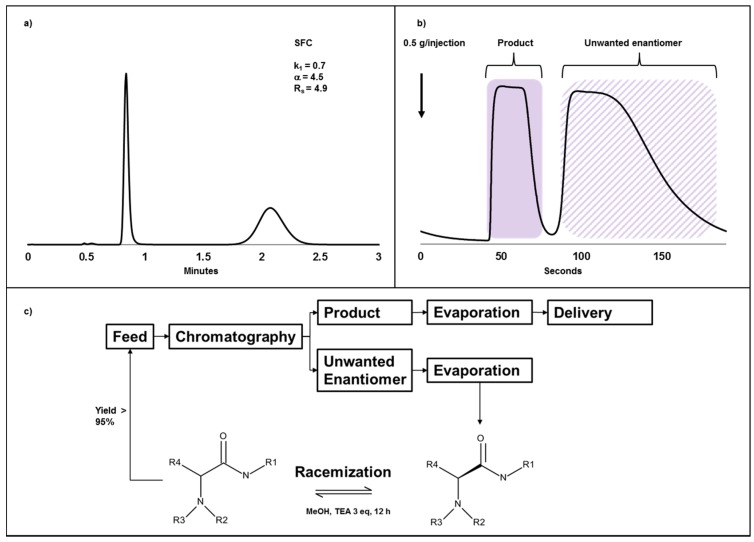
Chiral separation of compound **C**, a racemic amide. (**a**) Analytical SFC chromatogram using a Chiralart SA 150 mm × 4.6 mm, 3 µm column. Mobile phase was 40% 2-propanol (*v*/*v* %) in CO_2_ at 40 °C, 120 bar and a flow rate of 4 mL/min; (**b**) Preparative chromatogram on Chiralart SA 250 mm × 20 mm, 5 µm. Then 0.5 g (100 mg/mL acetonitrile) was injected every 190 s using 40% 2-propanol (*v*/*v* %) in CO_2_ at 40 °C, 130 bar and a flow rate of 80 g/min. The blue area corresponds to the product fraction and the dashed blue area corresponds to the unwanted enantiomer; (**c**) The feed solution (100 mg/mL acetonitrile) was injected and after chiral chromatography the enantiomers were collected and evaporated. The unwanted enantiomer was re-dissolved in methanol and trimethylamine (30 mg/mL and 3:1 molar equivalents to compound) and left overnight on rotation. The next day, full racemization had occurred and the racemate was re-used as feed solution.

Even though the synthesis including the separation of compound **B** at a kilogram scale above was successful in many aspects, it suffers from being uneconomical and wasteful since only 50% of the API was utilized after the separation step. It is, as discussed above, highly desirable to minimize the cost for reagents during the synthesis, and if the undesired enantiomer can be racemized and recycled back into the separation process, the costs can be minimized and the productivity greatly enhanced. For the initial toxicology evaluation of compound **C**, racemization of the unwanted enantiomer was an attractive alternative due to the limited access of starting material and extremely tight timelines. The compound was separated using chiral chromatography on a Chiralart SA column which is based on an amylose derivative, with a retention factor of 0.7 for the first eluted, desired enantiomer, and an α-value of 4.5 (Figure 3a,b) using 40% 2-propanol in CO_2_. The undesired isomer was found to be easily racemized under alkaline conditions due to the presence of the acidic hydrogen on the chiral carbon in α-position to the carbonyl group and our efforts to racemize the unwanted isomer gave good yields (Figure 3c). As a result, the last eluting and unwanted enantiomer was evaporated to dryness after chiral chromatography, re-dissolved in methanol and trimethylamine (3:1 molar equivalents to the compound) and left overnight to be fully racemized (>95%). After evaporation, the compound was re-resolved by chromatography, and after three cycles of chromatography and racemization, a total yield of 87% was reached. Further efforts to racemize and chromatograph were not pursued as a sufficient amount of the desired enantiomer had been obtained. The throughput for this separation was 4.4 g racemate/g CSP/day and scalable to kilogram amounts. The solvent consumption was higher for the chiral SFC separation of compound **C** compared to the previous case study (compound **B**) but significantly lower than using NPLC. The combination of chiral chromatography and racemization can be a powerful and efficient methodology to obtain pure isomers for biological testing in drug development at high yields.

## 3. Materials and Methods

### 3.1. Chemicals

All HPLC grade solvents were obtained from Sigma-Aldrich (Seelze, Germany) except for ethanol (99.5%) which was obtained from Kemetyl AB (Haninge, Sweden). Liquid carbon dioxide was purchased from AGA Gas AB (Stenungsund, Sweden). Diethyl amine p.a. (DEA) from Fluka (Buchs, Switzerland) and formic acid p.a. (FA) from Riedel-de Haën (Seelze, Germany) was used.

The compounds used were synthesized at AstraZeneca R&D (Gothenburg, Sweden) or at our partner contract research laboratories.

### 3.2. Instrumentation

Analytical chromatography: SFC runs were performed on an ACQUITY UPC^2^ equipped with a PDA detector and two column ovens with seven column positions each (Waters, Milford, MA, USA). HPLC runs were performed on a Waters 2695 consisting of a pump, an auto injector and a Waters 996 UV-detector (Waters). The chromatographic data was collected using Empower 3 Pro software (Waters) for both SFC and HPLC.

Preparative chromatography: SFC separations were run on a SuperSep 600 and the HPLC separation was run on a Hipersep LAB LC110 both using Proficy HMI/SCADA IFix 5.1 software (NovaSep, Pompay, France).

### 3.3. Chiral Stationary Phases (CSPs)

The columns used for screening were Chiralpak (AD, AS, IA, IB, IC, ID, IE and IF) and Chiracel OJ purchased from Chiral Technologies (Illkirch, France), Lux Cellulose 2 and 4 from Phenomenex (Torrance, CA, USA), Kromasil CelluCoat from Eka Chemicals (Bohus, Sweden), Chiralart Amylose-SA from YMC (Kyoto, Japan) and Whelk-O1 from Regis Technologies (Morton Grove, IL, USA). The dimensions and particle size for the columns were 150 mm × 4.6 mm I.D. and 3 µm in SFC mode and 250 mm × 4.6 mm I.D. 20 µm in LC mode. The preparative columns used for SFC were purchased as pre-packed from Chiral Technologies (Illkirch, France) and YMC (Kyoto, Japan). The 20 µm bulk material used in the HPLC column was purchased from Chiral Technologies (Illkirch, France) and packed in house using an LC110.700 VE70 DAC column from NovaSep (Pompay, France).

## 4. Conclusions

The preparative resolution of enantiomers using HPLC and SFC is a powerful technique for rapid separation of enantiomers in pharmaceutical research and development. The use of chiral chromatography for preparative-scale resolution of enantiomers has been shown to be particularly useful in the lead optimization phase to enable the selection of candidate drugs as well as for toxicology studies and first-time-in-humans campaigns. We have shown that while the broad applicability of chiral stationary phases allows the majority of racemates to be resolved routinely, there are some racemates that do not scale up as expected due to low solubility, poor loading or other unknown reasons. Further, the introduction of immobilized chiral stationary phases has enabled the separation of enantiomers using more unconventional solvents both in SFC and HPLC which would otherwise be very time-consuming and/or impossible due to poor compound solubility. When possible, utilization of carbon dioxide as the main solvent is preferable from a sustainability, health and economic perspective. The combination of chromatography, racemization and/or crystallization, as well as the informed choice of when to apply preparative chiral chromatography in a multi-step synthesis of a drug compound, can further facilitate accelerated drug discovery and the early clinical evaluation of drug candidates. Thus, to develop efficient synthetic routes for drug candidate compounds at a gram to kilogram scale, the experience from our laboratory strongly advocates a close partnership between synthetic chemists and separation scientists to develop the most efficient strategies for positioning the resolution step within an organic synthesis rather than utilizing chromatography as a rescue technology which continues to be the most common way of working in the industry.
